# Broad consent for biobanks is best – provided it is also deep

**DOI:** 10.1186/s12910-019-0414-6

**Published:** 2019-10-15

**Authors:** Rasmus Bjerregaard Mikkelsen, Mickey Gjerris, Gunhild Waldemar, Peter Sandøe

**Affiliations:** 1 Dept. of Food and Resource Economics, University of Copenhagen, Copenhagen, Denmark; 2 Danish Dementia Research Centre, Dept. of Neurology, Rigshospitalet, University of Copenhagen, Copenhagen, Denmark; 3 Dept. of Food and Resource Economics and Dept. of Veterinary and Animal Sciences, University of Copenhagen, Rolighedsvej 25, 1958 Frederiksberg C, Copenhagen, Denmark

**Keywords:** Informed consent, Biobank research, Autonomy, Consent models, Risks, Ethics

## Abstract

**Background:**

As biobank research has become increasingly widespread within biomedical research, study-specific consent to each study, a model derived from research involving traditional interventions on human subjects, has for the sake of feasibility gradually given way to alternative consent models which do not require consent for every new study. Besides broad consent these models include tiered, dynamic, and meta-consent. However, critics have pointed out that it is normally not known at the time of enrolment in what ways samples deposited in a biobank may be used in future research and that, for a consent to be *informed*, exactly this kind of knowledge is required. Therefore, there is an ongoing debate about the ethical acceptability of going for less than study-specific consent.

**Main text:**

In light of this debate we address the question of how to best protect participants against relevant risks and violations of autonomy. We apply the central aims of the informed consent process to the unique circumstances of biobank research where samples and data in many cases are stored for long periods of time and reused in subsequent studies. Thereby we are able to formulate a set of criteria focusing both on the risk of informational harm and the potential violation of participants’ values. We compare existing models of consent based on their ability to satisfy the criteria, and we find that the broad consent model offers the best level of protection for participants, although, it suffers from a few important deficiencies with regards to protection against participant value violations and long-term protection of autonomy, if it is applied without qualifications. For this reason, we propose modifications to the current broad consent model, in order to ensure that it provides protection of autonomy and participant values through strong ethical review and continuous communication.

**Conclusion:**

We conclude that a modified form of broad consent is ethically superior in biobank research, not only because it is most feasible but primarily because it offers the best available protection against the hazards facing research subjects in this form of research.

## Background

The emergence of biobanks as a vital research tool in the medical sciences has led to widespread debate in the literature about how to best handle the informed consent procedures governing the enrolment of participants in research, and the subsequent use of participant samples and data in other studies (see e.g. [1–9].

Before the ethical debate about biobanks emerged in the 1990s [10], study-specific informed consent (wherein consent is obtained from all participants immediately before each new study is undertaken, and the consent only covers the specific study in question) was the norm in human subject research, and it became the foundation of international as well as national research ethics policies [11–13]. This consent was designed to ensure that participants were well informed about the goals of the research and about any relevant risks or benefits of participation. However, the practicalities of biobank research (in particular the size of the cohorts and the frequency of new studies) have made it difficult to replicate study-specific consent in biobanks. This has led to the development of new and adapted consent procedures that aim to overcome these difficulties while retaining the protection of participants that informed consent offers (see e.g. [7, 8]).

To date, different versions of the broad consent model have generally been adopted by those managing biobanks (although there are significant variations in the implementation of informed consent across countries, based on both culture and legislation). When this model is applied, general consent is gathered at the time of enrolment (subject to a set of limitations and restrictions that are formulated by the biobank and/or a regulatory authority and stated in the consent form). Subsequently, samples stored in the biobank can be used for new studies that fall within the scope of the consent without re-obtaining consent from participants. Proponents defend the broad model by arguing that it is the best way to make large-scale biobank research feasible. However, critics have pointed out that it is normally not known at the time of enrolment in what ways samples deposited in a biobank may be used in future research and added that it seems that, for a consent to be *informed*, exactly this kind of knowledge is required [6].

In this way, arguments often revolve around how different models of consent measure up against one another and the extent to which each succeeds in offering the same level of protection as the traditional forms of consent known from clinical research. In this paper, we suggest that this type of comparison is of limited use. We argue that study-specific consent has rightly fallen out of favor in biobank research precisely because the context and structure of the latter differs significantly from that seen in the studies for which study-specific consent was originally designed. We conclude that the traditional consent model does not offer a useful basis of comparison, and hence does not allow us to gauge the quality of informed consent models in the biobank context.

Study-specific consent procedures are effective in informing potential participants about the particular, often physical, risks posed by a specific study. Plainly, these are the most important risks to consider in traditional human-subject clinical research. However, after the procurement of the initial sample and its allocation to the biobank, physical risks to participants are minimal. On the other hand, the risk of informational harm from leaks or hacks, or violations of a participant’s values as a consequence of the types or methods of research carried out in the biobank, are correspondingly larger. The likelihood of these harms, moreover, is dependent on the governance and policies of the biobank, rather than the way in which any given research project is conducted. The purpose of informed consent (which will be explored in more detail below) is to inform participants about the relevant risks and benefits of participation and the goals of the research, and to allow participants to exercise their autonomy. Study-specific consent was designed to inform participants about study-specific risks of research projects, because historically these were the clearest risks of participation in traditional research projects. However, the risks associated with biobank research are quite different and more general, and for this reason it cannot simply be assumed that the study-specific consent model will provide suitable protection for participants in biobanks. We argue that because the risk profile has changed, a different model of consent structured to address the relevant risks is needed for biobank-related research.

In this paper we argue that a modified version of broad consent best serves to protect participants in biobank research, based on the novel criteria-based model of assessment of consent models presented in this paper. We begin, in the next section, by providing a general overview of the main models of informed consent and the principal criticisms that have been levelled at them. We then turn to the aims of the informed consent process and discuss how these aims apply to biobank research. Drawing on this discussion, we formulate a set of criteria designed to ensure that the *aims* of the informed consent process are met in the context of biobanks. This allows us to evaluate models of consent that have been presented in the literature by this novel standard. We conclude that, as long as it is combined with strong institutional protections and provides long-term protection for participants, the broad consent model is best suited to meet the stated aims of informed consent in biobank research. In other words, the broad consent model is best, provided it is also deep.

## Proposed models of informed consent

### Study-specific consent

Initially, it would seem that the most straightforward way of ensuring that the aims of informed consent are met in biobank research is to implement best practice in consent procedures, of the kind found in traditional human subject research. This would mean that informed consent is obtained before the commencement of each new study, and that the consent is specific to the risks and benefits of the study being proposed. Thus, in this model, samples contained in the biobank may only be re-used in new studies after consent has been obtained for that particular use either by the participant or, in cases where that is not possible because the participant has for one reason or another lost decision-making capacity, an appropriate surrogate. This would allow potential participants (or their surrogates) to evaluate whether they wish to participate with reference to specific information and ensure that any agreement to participate respects the autonomy of the participants to the greatest extent possible.

However, implementing study-specific consent in biobanks is problematic. The structures and processes involved in biobank research are vastly different from those in clinical research. Consequently, the types of participation and interaction study-specific informed consent was designed to address are not always present in the biobank case [14]. There are three general differences between traditional human subject research and biobank research: First, samples and data stored in biobanks will potentially be used in new research projects dozens, or hundreds, of times a year. Second, biobanks often store samples and information over long periods of time (years or decades). Third, where biobank research is concerned, participants are recruited and samples are gathered before most of the future research has been planned or even thought of. While these structural changes have enabled new kinds of research to be carried out – as seen, for example, in Surén et al.’s study of the relationship between the use of folic acid supplements during pregnancy and autism spectrum disorders in children [15] – they also challenge the traditional, study-specific model of informed consent. The study-specific consent model requires researchers to obtain consent to use already collected samples whenever a new study is begun. However, much of the benefit of biobank research depends on researchers’ ability to reuse samples in many future studies. With this in mind, it can readily be seen that the study-specific consent model is liable to limit the utility of biobank research in general (assuming that another consent model involving less frequent consent is ethically feasible) both because it requires resources that could have been used for research to be devoted instead to the acquisition of later consents and, not least, because failure to receive new consent from part of the study population may give rise to various biases. Moreover, there is a risk that repeated requests for consent will lead either to consent fatigue, or to routinization, resulting in turn either in fewer participants, or in watered-down consent [8, 16].

### Broad consent

Confronted with the challenges facing the study-specific consent model, many biobanks have opted for a broad consent approach [17]. Broad consent, as understood here, is consent obtained at the time of enrolment in the biobank against a background of assurances about the overall scope and aims of the biobank as well as its governance [18, 19]. Subsequently, samples and information can be reused without obtaining a new consent, as long as the use falls within the scope of the original broad consent and also fulfills other regulatory requirements such as approval from an ethics committee.

Having been informed about the general scope of the biobank, participants are able to evaluate whether they wish to consent to the overall biobank policy – and, by extension, to the types of research that the biobank permits upon any samples and information the participant has provided. However, critics [6, 7, 20] have argued that this model does not afford the same level of protection as study-specific consent, and that it is simply a pragmatic way to allow research to progress without informing participants about the specific risks and benefits of future studies that have not yet been planned or even conceived. Furthermore, in biobank research incidental findings may be made, or intended research results may lead to information, which could be relevant to the health of a unique participant. Within a broad consent model, it will generally be difficult, if not impossible, for researchers to foresee these situations. This means that it will not be possible to allow participants to make specific decisions about the return of their individual research results when they sign the consent form – something that is possible, of course, in the study-specific consent model.

### Dynamic consent

Problems like those described above have led several authors to suggest new or adapted models of consent that are specifically designed to meet the challenges of biobank research. One of these is the dynamic consent model. This seeks to harness the potential of modern IT and communication systems to develop better procedures for obtaining informed consent to biobank research. The idea is to develop a digital platform which allows participants to access and modify their consent preferences continuously (including withdrawing consent), as well as track, and follow up on, the activities of the biobank [20].

The dynamic model offers a less cumbersome way to obtain study-specific consents, since the digital interface ensures that face-to-face contact is not necessary for each renewal of consent. With this advantage, it may be viable to enact study-specific consent procedures for each study that a participant’s information or samples are used in, even when the samples are being used in new projects several times a year. Kaye et al. [7] have argued that alternatively the platform could be set up to allow participants to choose to provide broad consent if they wish to do so.

The challenges facing the dynamic consent will be discussed in greater detail later in the paper (see Section 4.2). For now, we note merely that it is unclear whether this model is capable of securing the enhanced levels of autonomy obtained in the study-specific consent model without also encountering some of the issues the latter faces.

### Other models

A fourth model of informed consent is “tiered consent” [3, 21]. Tiered consent procedures allow potential participants to at least partly tailor their consent preferences around general categories. The model retains the underlying structure of the broad consent approach, in the sense that, in it, consent is provided for future research in general rather than specific research projects. However, the participant is able to be more specific about the uses that she is consenting to. Wolf and Lo found that the specific tiers offered by Institutional Review Boards (IRB) vary widely [21], but typically tiered consent procedures cover specific diseases or categories of diseases (e.g. cancer research), public/private research, identified/anonymous use, defined areas of research, or certain research institutions or specific researchers [3]. The tiered consent model is meant to allow participants to retain a greater degree of control over the use of their samples and data. It is also meant to be free of the problems associated with study-specific consent described above.

The meta-consent model shares many features of the tiered consent [8]. The key difference is that, while the latter only allows the participant to provide general consent to elected tiers of research, the meta-consent process is more flexible in that it allows participants to associate different kinds of consent with different tiers. For example, a research subject might decide that she wants to give study-specific consent to all future research that falls within the tier of “dual-use” (i.e. research with additional military applications), but that she is also happy to provide a single broad consent to all future publicly funded public health research. Meta-consent does not only allow participants to provide consent preferences regarding different categories of research, however. It also enables them to tailor their consent preferences to specific categories of data – as they may wish to do, for example, if they have special concerns about genomic data. Participants can therefore design consent portfolios which require them to make specific decisions only about certain types of research.

While both the tiered and meta-consent models are promising, in that they allow fine-grained participant control without requiring consent for every study, they face two obvious challenges. The first is that their effectiveness relies on the competence of the participants, and specifically their ability to understand the possibilities, and to construct a consent portfolio that is acceptable to them. To be specific, while all consent models must address differences in competency insofar as this affects potential participants’ ability to provide informed consent, the tiered and meta-consent models are doubly vulnerable to this issue. This is because participants’ competency will not only affect their ability to provide consent, but also the quality of the consent procedure itself. The second is that tiered or meta-consent will often require projects to be categorized in a way that is not practicable. The main issue here, which will be discussed in more detail in Section 4.3, is that research only rarely falls into neat categories of e.g. private/public or national/international, which would allow the type of categorization that is necessary to allow fine-grained participant control.

### Comparing the models

The models of informed consent presented above all appear to solve some of the issues that have been identified within the broad and study-specific approaches to informed consent, but we have also explained that, prima facie at least, each contains challenges. From our perspective, this is because they seem to be attempts to treat the symptoms of a disease that has not yet been diagnosed. Without this diagnosis, it is difficult to say whether the adapted models can successfully solve the problems posed by the consent process in biobanks.

To provide the necessary diagnosis, and assess the adequacy of the models, we will need to be clear about the purpose of informed consent to participation in research projects. This purpose is captured in two main aims. Informed consent aims to:

- Inform potential research subjects about the relevant risks and benefits of participation, and the goal of the research;
- Allow potential participants to exercise their autonomy by accepting or refusing to participate

These aims should be interpreted broadly and include information about direct health risks as well as information about data storage and access policies affecting informational risks, and the intended goal of the research including financial and commercial interests. The aims have been central to the development of informed consent in the research context and are contained (by implication at least) within the major guidelines on informed consent for human subject research around the world [13, 14, 22]. However, it remains an open question how the aims can be met in biobank research. To answer this question, it is necessary to provide a clearer picture of the structures and processes within biobank research which have implications for the ethical protection of participants.

## Relevant criteria for informed consent to Biobank research

Obviously, in providing the most accurate information to potential research subjects, the person seeking consent should focus on information about risks that are relevant to the project in question. The consent process should not address the risk of being hit by a bus going to the test center, for instance, since that risk is not especially large; nor is it particularly associated with the proposed research (unless, of course, the test center is located in an area prone to bus accidents). In general, the consent process should focus on informing candidate subjects about potential harms that are sufficiently severe and sufficiently likely to be realized. This will give them the most accurate understanding of the risk profile of the project they are considering participating in.

In the informed consent process followed in traditional medical research, the scope of things to consider is mostly fairly straightforward: The important issues under consideration are typically the risk of one or more physical or psychological harms as a result of a physical act or intervention that is part of the research protocol [23]. For example, a study examining the effect of regular treadmill exercise on muscle mass and composition might identify the relevant risks as the risk of injury caused by physical exertion, the risk of pain or infection associated with potential biopsies of muscle mass, and so on.

### Informational risk

Biobanks will often hold information about individual participants, including information from medical records, along with biological samples and/or genetic information, for long periods of time. This means that informational risk – i.e. the risk of harm resulting from the revelation of personal information, including genetic information, through hacks, leaks, or unintentional distribution – can be of great importance to the individual enrolled in the biobank.

Two general contributory factors are involved in this risk: First, biobank research carries with it an incentive to gather as much data as possible on participants because at the time of procurement there is often no set use for the data. (This is especially true for non-specific disease biobanks.) The basic idea behind biobanks incentivizes broader, and more intensive data collection, and the retention of all data, independently of the dataset’s current usefulness. The effects of this incentive can be seen in recent efforts to combine databases across biobanks in order to achieve even larger databases and more ‘fine-grained’ data, and in the policy of some biobanks of continuing to gather and update information in the biobank through links to electronic medical records and the like for the duration of participation [12]. Second, while stringent security protocols might be observed, the simple fact that personal and potentially sensitive information is gathered, stored within and shared by the biobank for long periods of time will put the information at risk.

But it is noticeable that their likelihood of being actualized depends more on the governance, policies, and competence of the biobank than it does on specifics of the research being carried out. It remains the case that participants will be put at risk if samples and data are shared with researchers who have insufficient data and sample security practices. However, the likelihood of participants being put at risk in this way in large part depends on the distribution policies of the biobank, and the agreement it has in place with researchers obtaining access to samples and data. Furthermore, risk also attaches to the storage and distribution policies of the biobank as such, and the combination of data from many sources significantly heightens them [24]. Study-specific informed consent procedures are not equipped to deal with such risks, since the risks cut across the specific studies.

### Potential for violations of participants’ values

Another novel issue that biobanking raises turns on the potential transgression of participants’ values. Because biobanks often provide samples and data to many different research projects every year, a research project approved by broad consent might end up contradicting the values of some contributors to the biobank. This issue is especially likely to arise in biobanks where participation stretches over long periods of time. First, the values of the participant may shift over time, with the result that the values that led her to provide consent are abandoned or superseded by others. That may mean that research that is initiated may violate the participant’s values at a later time. Second, as research methods and aims develop, new research projects may be proposed which neither the participant nor the biobank administrator envisaged at the time of enrolment, thereby making it difficult, or impossible, to know whether a specific research project is objectionable to some portion of the cohort. Even broad guidelines given at the time of enrolment may well not be sufficient to protect individuals from this risk of violation of their values.

### Criteria for informed consent in biobanks

In the literature there has been a tendency to judge consent models applied to biobanks on their ability to afford the same level of protection as traditional, study-specific consent (for examples, see [2, 6, 20]). However, as we have explained above, the types of protection needed in biobank research differ markedly from the security traditional consent models are designed to offer (which relates chiefly to known, specific, and often physical risks associated with a particular study). Thus, any fair assessment of a consent model for biobank research must be based on criteria that are relevant to this particular kind of research, rather than on a comparison with existing models. We propose three criteria that the consent model should fulfill:

1. **Information Criterion**: Inform potential participants about the relevant risks and benefits of biobank research (especially informational risk) so that she can make an autonomous choice about participation in light of this information;
2. **Value Criterion:** Offer participants the opportunity to assess whether the research to be conducted with biobank materials is in line with their personal values, and to make consent decisions based on this
3. **Duration Criterion:** Afford ethical protection for the duration of the subject’s participation in the biobank, and give the subject a real and meaningful opportunity to reassess her consent for that duration and an actionable right of withdrawal (provided, of course, that the participant is still alive and competent).

The information and value criteria are a direct consequence of the analysis of the relevant risks of participation in biobank research, as described above. Notably, the information criterion is formulated as it is in light of the fact that the risks involved in participation in biobank research depend largely on features of the biobank itself, rather than on the specifics of any given study.

The duration criterion is of special concern in biobank research. While a right to withdraw has been standard in informed consent procedures for many years, biobank research requires more than just stating the availability and terms of such withdrawal. Participation in biobanks takes place over long periods of time, without the direct participation of the individual. This means there is a need to put in place ongoing ethical protection in order to ensure participant autonomy, as there is a greater risk that subjects will forget that they are participants, or simply cease to think about their involvement [5]. This problem is especially challenging for broad consent models, because where they are applied there will normally be very limited, or no, continuing communication with participants. Thus, it is more likely that participants will be unaware of, or will have forgotten about, their participation and their right to withdraw.

Fulfilment of the three criteria should ensure that an informed consent model is successful in providing consents satisfying the aims of informed consent and affords adequate protection for participants. This means that the models of informed consent that have been proposed for adoption in biobanks can be evaluated against these criteria in order to determine whether any of them provides superior protection for participants.

## Evaluating proposed consent models against the criteria

We will now test the main models of informed consent against the criteria set out above.

### Study-specific consent

Study-specific consent would require biobanks to obtain consent for each study. Clearly this would have a substantial impact on the ability of biobanks to carry out research, and would place a particular burden on disease-specific biobanks that work with end-of-life patients or competence-limiting diseases such as dementia. This issue has been discussed in the literature previously, especially by proponents of broad consent [4, 23], and dynamic consent has been presented, in part, as a solution to it [7]. As a consequence, the study-specific consent model has generally not been adopted in biobanks.

Study-specific consent probably also fails to meet the information criterion, as it is structured to focus on information about the risks of a particular study, while the relevant risks in biobanks, as explained above, are a consequence of general biobank governance, policy, and competence. This means that a consent process with focus on specific uses of samples is not geared to offer participants relevant protection.

The maintenance of adequate study-specific consent would also be likely to require such frequent efforts to obtain separate consents that a balance would have to be struck between the demands of consent and the resources available to those charged with obtaining each consent. The former may well have to be traded off against the latter, with corners being cut. This could put the quality of an individual’s consent at risk, especially since there is some evidence that understanding is improved by face-to-face contact in the consent process [25, 26], which is a resource intensive process. In all likelihood, then, the consent procedures for secondary research on samples would end up being administered without face-to-face contact. There is good reason to believe that the quality of the consent would be poorer as a result.

### Dynamic consent

If, as suggested by Kaye et al. [7], dynamic consent is employed as a delivery system for study-specific consent, it must initially be assumed that it will also suffer from the problems besetting study-specific consent just discussed. However, this does not exclude the model from consideration, since dynamic consent can be used to deliver many different forms of consent, as its proponents have pointed out [7]. In theory, dynamic consent may, in effect, ‘drift’ between consent models, and thereby inherit the benefits of each, depending on the context.

However, in practical terms, the dynamic consent platform must deliver consents based on some procedure or other, and that procedure should be based on a defined and recognized method of consent (like the methods described in the other consent models) in order for the platform to function in an acceptable way. In this case, it must be assumed that when the dynamic consent platform is used to deliver study-specific consent, both the benefits and costs of the study-specific consent model apply. This will be the case unless there is some special feature of the dynamic consent, or the use of a particular consent model within this platform, that ensures that a certain cost does not accrue in the case at issue.

Thus, if it is suggested – as it is, for example, in Kaye et al.’s paper [7] – that dynamic consent has the advantage that it promotes autonomy as a consequence of its ability to deliver study-specific consents, then it must also be acknowledged that, prima facie at least, the costs associated with the study-specific consent model are nevertheless a drawback. While supporters of the dynamic approach can rightly claim that, with its mode of delivery, it is capable of offsetting some of the inefficiencies of the more traditional study-specific consent model, they must also accept that at least some of the challenges posed by study-specific consent (of the kind touched upon above) must also be addressed: This is inevitable, given that the dynamic model must, in reality, commit to a particular consent model in order to function.

In addition, the model suffers from a unique weakness: It runs the risk of failing to protect the autonomy of the participants sufficiently, as dynamic consent is unable to ensure that potential participants make decisions about participation that are based on a high level of information and understanding without external influence or coercion. This issue is relevant to, and needs to be addressed in, all of the consent models presented in this paper, but the dynamic consent procedure is particularly vulnerable to it because it does not enable adequate protections to be put in place. Dynamic consent intentionally differs from traditional consent by allowing participants to provide consent in uncontrolled conditions. Traditional consent procedures emphasize the minimization of undue influence and coercion by placing controls on the consent environment (e.g. by ensuring that consent is provided in private, without time pressure, and with the oversight of a professional who can assess the competency and mental state of the participant to an extent). In practice the digitally administered consent procedure *might* be able to screen for people with mental disabilities or other impairments that are registered in public health records, but it cannot ensure to a reasonable degree that potential participants are not subjected to undue influence. In the simplest terms, this might mean that a potential participant is in an environment – e.g. in the presence of a friend or family member who holds certain values dear or has an interest in the person making a particular decision about specific areas of research – where she feels pressured to make decisions she would not otherwise have made.

### Tiered/Meta-consent

The tiered and meta-consent models provide categories of context, research methods and aims, and types of data which allow participants to be aware of the specific tiers on which their samples and data can be used, and to withhold consent from uses that are not in line with their values, while continuing to participate in the biobank. However, these models rely on the ability of those running the biobank to draw relatively clear definitional boundaries around the different categories. It is not clear that this aim is as easily achieved in practice as it is in theory, especially as new research areas and methods emerge over time.

Ploug and Holm [8], who advocate the meta-consent model, suggest ‘private’ and ‘public’ as potential tiers that can be used to differentiate between consent standards. However, today much (if not most) of the medical research being pursued in public institutions is conducted in partnerships with private stakeholders. The partnerships range from simple knowledge-sharing, or consulting, all the way to fully integrated collaborations involving shared patent rights. In other words, modern research does not offer a simple dichotomy between public and private research, or for-profit and not-for-profit research, and to offer such a choice between these categories would be inefficient at best and misleading at worst.

Given this, biobanks will be obliged either to deny sample use in all borderline cases (which would hamper research progress) or to make judgments internally without the input of the participant who provided the consent (which would water-down the consent). Thus, on closer inspection meta-consent seems to not have the fundamental advantage over broad consent claimed for it: It does not, in other words, offer the participant a greater degree of control. It seems that the tiered and meta-consent models aim to give participants a more well-defined and finely tuned web of categorization than is practically possible. In this way, the models fail the duration criterion, as developments in the methods and aims of research will diminish the level of protection provided by earlier consents over time.

Even if a solution to this problem of emerging, or simply changing, research focus is found, the practical implementation of the models is bound to leave a lot to be desired. The hope that a tiered or meta-consent model would allow participants to exert greater control over the way their samples and data are used without resort to a study-specific consent model (along with its associated issues) is unlikely to be fulfilled. The model requires repeated re-categorizations of research and associated re-consent procedures, and this points to a larger underlying nexus of issues. The tiers involved in the models do not by themselves represent values, but rather categories of research. This creates several problems. First, potential participants will make decisions about the categories they wish to consent to (or, in the case of meta-consent, about which model of consent they would like to be applied to a specific category). Their decisions will be based on their underlying values, together with their beliefs about the category, or categories, in question. However, these values are not explicitly recorded against the categories. As a consequence, it will not be possible for the biobank administrator, or user, to know what value judgments led a person to provide consent for a specific category. By extension, administrators and users will be in no position to assess what changes to the boundaries of the category can be made without stepping beyond the expectations under which the participant’s consent was originally given.

Imagine a participant has provided consent for her samples and data to be used in cancer research. She has done so because she believes that cancer research is valuable, but also because she assumes that this type of research will not involve the cloning of human tissue – a restriction which, for her, is a condition of consent. If at a later time human tissue cloning becomes an important technique in a certain type of cancer research, the biobank administrators will be likely to make her samples available for this kind of research, because they have no record of the underlying values and information that led the participant to give her consent originally. Unwittingly, the biobank would involve the participant in research that is contrary to her values. In this way, the tiered or meta-consent models may fail the value criterion, because the boundaries of specific categories will generally be based on a highly uncertain interpretation of the likely values and beliefs that would guide participants’ decisions about those categories.

Lastly, it should be pointed out that attempts to devise a set system of categorization is bound to reflect a set of values – values which define the categories of relevance, their boundaries, the tiers, and so on. This raises a concern about the fairness of tiered and meta-consent, since potentially it means that minority groups with alternative value sets may not enjoy the same level of control and protection from these models as the majority group would (see [5] for some empirical work on the way demographic and other factors affect people’s perceptions of biobank research, and their willingness to participate).

### Broad consent

Having identified a number of problems with the study-specific and adapted models, we turn now to consider broad consent. This model has been criticized for not living up to the standards of informed consent [6]. The complaint is that it fails to provide potential participants with enough information, or information of sufficient depth, about the specific research projects for which they will provide samples or data. If this were correct, it might be concluded that the model fails the information criterion. However, in light of the capacity of broad consent to identify the relevant risks of participation in biobank research, it can be argued that this model is actually particularly well-placed to meet this criterion, as the main risk of participation in biobank research is informational risk.

Importantly, this risk is mainly a consequence of having potentially sensitive personal information placed in the biobank and stored there for long periods of time. Further, the likelihood of the risk being actualized is mostly a consequence of the governance, competence, and policies of the biobank [10]. Consequently, it is entirely possible for the broad consent process to cover relevant information about the informational risks of participating in the biobank, and to describe the structure and governance of the biobank at the time of sample collection, and thus inform candidates about the relevant risks of participation.

By extension, broad consent is also effective in supporting the potential participant’s autonomy. The structure of the consent sought here encourages participants to reflect on the general risks of informational harm that the biobank creates as a whole, rather than on the specific risks associated with any given research project. Additionally, when consent is structured as a single consent, biobanks can provide a much deeper consent process by investing more resources in the practical process of consent at the time of enrolment, which facilitates greater investment in face-to-face time for participants. This lowers the risk of uninformed decision-making and undue external coercion that features in some other models of consent. Moreover, the broad consent model has a less severe impact upon the ability of scientists to carry out research within the biobank. Overall, then, broad consent appears to successfully support both the information criterion and participant autonomy while promoting the efficient use of resources.

#### Drawbacks of broad consent

However, this leaves the value criterion and the duration criterion. By providing broad consent at the outset, participants will gain some protection against their samples being used in research that is contrary to their values, but that protection is rather shallow, since the broad scope of the consent will probably not include enough detail to ensure that all proposed research projects clearly fall on one side of a boundary or the other. It is also a concern that, as research aims and methods evolve over time, it is unlikely that the parameters of the broad consent obtained will readily apply to all of those aims and methods. There is a real risk that some participants will end up participating in research that they would not have agreed to, if they had been asked for a study-specific consent.

Broad consent may also reinforce the idea that ‘ethical work’ is concluded when the consent has been obtained. This is of course a general misconception, not one that the broad consent model uniquely invites, and therefore it should not be seen as an argument against the model. However, it might be said that even if the broad consent model is not unique in being exposed to this misconception, it might be more likely to occur when this model is used. It might be added that, all else being equal, a model which makes this mistake less likely (e.g. a study-specific consent model) is superior in this regard.

If participants are to receive relevant protection over the duration of their participation, their values must continue to be considered throughout the life of the biobank. If participants provide a broad consent at the time of enrolment but are not aware of future developments in the work of the biobank, they are unlikely to be fully aware of the scope of research they are enrolled in, which means that the value of their right to withdraw is questionable. To be ethically acceptable, then, the broad consent model needs to be deepened.

## A proposal for a broad consent model that is also deep

Broad consent as it stands does not provide adequate protection. It fails to satisfy both the value criterion and the duration criterion. However, we believe that these challenges can be addressed and to some extent met. In essence, we propose that two elements are needed in order to ensure that the broad consent provides the required level of ethical protection for participants: A strong and continuous ethical review process, with the specific mandates for proposed research resting on evaluations of whether that research falls within the scope of the broad consent, and continuous provision of information to participants.

### Ethical review

An ethical review must be mandated and guaranteed if broad consent is to serve its purpose. Earlier broad consent proposals have relied largely on existing ethics reviews to provide the ethical assessment of particular studies, given the broad consent provided by participants. However, as participants are not able to assess each new study in accordance with their values, an additional step in the review is necessary. Independently of other ethical review questions, a study intending to use data from the biobank should be assessed by the biobank administrators (or in relevant cases by national/regional ethics boards) to determine whether it falls within, and complies with, the boundaries of the broad consent that participants have provided. Whether this can be done within existing structures will depend on the setup of the biobank and the context in which it operates, but it should be explicitly stated that this step of the review is both mandatory and independent of other ethical and practical considerations when studies are being approved. Thus, the following requirement is essential for the ethical approval of a new study undertaken under an existing broad consent: It must have been found to be within the scope of the broad consent as judged by the relevant kind of ethical review.

It will be necessary for the biobank to clearly set out the conditions which qualify a study for this extra level of ethical scrutiny, although these conditions will depend on both the features of the biobank and the population it contains. In setting out these conditions, the biobank must be mindful of potential minority views or values which might be violated, even if the study remains uncontroversial for the majority of participants. The review needs to have the sole aim of establishing whether the proposed research falls within the scope of the broad consent that has been obtained from participants. In this way, the review will ensure that participants’ values are considered in every decision without the need to re-contact them.

For this process to be effective, the original broad consent must involve clarity about the scope of the biobank. This requires the broad consent to identify the core values that will guide decision-making about future research projects as well as any potential research areas that are excluded from the scope. The increased level of specificity within the scope of the broad consent, which is necessary to ensure the proper functioning of the ethical review, will create a certain degree of inflexibility in terms of what types of research can be undertaken using the information and samples held by the biobank. Unfortunately, this requirement will create both intended and unintended restrictions on future research projects which could have been approved under a study-specific consent approach, and it is to be expected that with developments in research aims and methods this issue may be exacerbated over time. However, without this clearly defined scope, the protections provided by the ethical review are fundamentally undermined, and for this reason this is a necessary concession within the broad consent model.

It must be emphasized that evaluations will be made without considering the opinions of each participant, creating some level of risk that a participant would disagree with an evaluation, especially in borderline cases, and thus the possibility of participants being unwittingly enrolled in research that is contrary to their values. While this is an undesirable consequence of the model, it should be clear from the above that no model except the study-specific consent model can provide a higher degree of protection from this risk. Since study-specific consent suffers from several other issues that make it an unattractive candidate for implementation, and since broad consent remains at least on par with all other models on this issue, the latter could be regarded as the least problematic alternative. It is also worth noting that this issue can be further addressed (although not completely resolved) through the implementation of continuous information for participants.

### Continuous information

By continuous information we mean regular communication with all participants in the biobank who are still alive and competent describing the work that has been done within the biobank since the last communication and giving information about future plans to the extent that this is possible. This must include information about notable results from recent studies as well as newly approved research. It might also contain more general information about developments within the field of the biobank, and about how these relate to the operation of the biobank. This information should be provided at defined, regular intervals (e.g. once a year). While many biobanks provide information of this or a similar sort of their own accord, we propose that the provision of continuous information should be mandated within the broad model of informed consent suggested here. It would no longer simply represent best practice. Instead it would become a necessary condition of compliance with the consent given. Specifically, it would be stipulated in the consent form that potential participants are asked to sign that the validity of any consents they provide is contingent upon the biobank providing continuous information meeting the standards described here (taking into consideration the context of the particular biobank and the implementation of the model of consent suggested here).

Providing this type of information has many benefits. First, it allows the biobank to build trust with participants. There is evidence that such trust is central to willingness to participate [27]. Second, continuous information allows participants to keep abreast of the evaluations made in the ethical review process. This ensures that they are aware of the research that is taking place and allows them to evaluate whether their values continue to align with the wider activities of the biobank and consider whether they still wish to be enrolled. This, in combination with the continuous review process, offers a further safeguard which reduces the possibility of participants being enrolled in research that is contrary to their values and thus meets the value criterion.

Third, continuous information ensures that surviving participants who are still competent are always aware of their participation and their right to withdraw. In this way, such information also serves to ensure that participants have a meaningful right of withdrawal at any time – which is a central concern of biobank research, as argued above – thereby satisfying the duration criterion. Thus, by thinking about the broad consent model in a wider perspective, and by being clear about the necessary criteria that informed consent must fulfil in the biobank context, it is possible to ensure a very high degree of fulfilment of all three of the criteria we have set out. This is important since, as far as we have been able to determine, the current, narrower descriptions of broad consent neglect the value and duration criteria.

The provision for continuous information is an important element in ensuring that broad consent satisfies the criteria set out earlier in the paper. Could it, however, lead to problems similar to consent fatigue? That is, would participants who are regularly presented with information in an intrusive and uninvited manner develop “information fatigue”, and would this lead them eventually to opt out of the biobank? It cannot be denied that this problem may affect enrolment in the biobank, but two important points should be considered before we throw the baby out with the bathwater. First, while it may be possible to address the problem by providing information only when relevant changes are made, or only when information matches the participant’s preferences, there is a worry that the strategy would raise its own issues. Thus, if participants prefer minimal information, communication could become so infrequent that the information provided then fails to satisfy its purpose in terms of ensuring awareness of participation. Also, any attempt to demarcate when “relevant”, or “important”, changes have taken place would have to rely on general assumptions about participants’ preferences and motivations that would fail to ensure equal protection for all participants. Second, it is arguable that any risk of information fatigue of the kind we are envisaging is unlikely to be as serious as the risks associated with consent fatigue. Continuous information allows participants to be as involved in the biobank as they wish. While it offers information to participants that are concerned about, or interested in, the work of the biobank, it does not actually require the participant to engage with the information. If a participant is content to participate without reviewing the information, this is a genuine option. By contrast, repeated study-specific consent requests do require participants to engage with the biobank, and to make decisions on a regular basis, irrespective of their wishes.

## Conclusion

The implementation of satisfactory informed consent processes in biobank research has proven difficult for both practical and ethical reasons. The structure of biobank research has forced a rethink of study-specific consent, and newly proposed, alternative models have been challenged on the sufficiency of their protections. In this article we have examined the most frequently proposed models and noted their major alleged weaknesses. Following this, by investigating the basic aims of informed consent as well as the unique challenges that are presented by the structure of biobank research, we have formulated a set of criteria for informed consent in biobanks against which the various biobank consent models can be evaluated.

The criteria reflect the fact that the risks traditionally considered most important in discussions of informed consent – namely, those of direct physical damage – are less important in the context of biobank research: Here, risks of informational harm and the violation of participants’ values matter most. The criteria for informed consent in biobanks suggested in this paper are therefore based on the idea of managing these potential risks. Given the criteria, and their focus on the unique risk profile of biobank research, we find that broad consent achieves the underlying aims of the informed consent process better than any other evaluated model. It is able to provide information about the relevant risks of participation, as these are, to a large extent, a consequence of more general facts about the biobank and its scope rather than any given research project. Broad consent is also effective in ensuring participant autonomy: Its structure encourages participants to focus on relevant information, and since consent is only gathered at the time of enrolment, biobanks are able to expend the necessary resources on creating an environment and information exchange that encourages autonomous decision-making.

We have argued that the broad consent model can only be an adequate model of consent if it is also deep. We have claimed, however, that the necessary depth can be added by thinking about the consent process in a wider perspective, ensuring that any consents obtained are based on sufficient information that is given to participants over time, and putting in place an effective right of withdrawal facilitated by that continuous information provision.

Broad consent is not a perfect solution to the problem of informed consent in biobanks. Even with the deep nature of the modified broad consent process set out here, there is still a risk that participants’ values will be inadvertently violated by future research. Furthermore, the necessary ethical review process requires the scope of the biobank to be defined fairly rigidly, and it is a foreseeable consequence of this that some future research that would have been allowed under alternative consent models might not be possible under the model we have proposed. However, while both of these drawbacks are undeniable, in our view the broad and deep model we have set out is a better model of consent for biobanks than any of its competitors. It is best suited to deliver participant protection while achieving the research aims of the biobank.

## Data Availability

Not applicable.
